# The hallmarks of host-microbiome decoupling

**DOI:** 10.3389/fmicb.2026.1801100

**Published:** 2026-04-14

**Authors:** Jhommara Bautista, Andrés López-Cortés

**Affiliations:** Cancer Research Group (CRG), Faculty of Medicine, Universidad de Las Américas, Quito, Ecuador

**Keywords:** circadian-host microbiome desynchronization, host-microbiome decoupling, immune miscalibration, microbial metabolite signaling, microbiome functional resilience

## Abstract

The human host and its resident microbiome maintain continuous interactions that influence immune regulation, metabolism, neuroendocrine signaling, epithelial barrier function, and circadian organization. Although multi-omics approaches have improved mechanistic understanding of host-microbiome interactions, dominant translational models remain largely based on compositional descriptions and often do not capture persistence, systemic propagation, or temporal instability in microbiome-associated disease. Host-microbiome decoupling is defined here as a progressive reduction in functional coordination between host regulatory systems and microbial ecological behavior. The concept refers to conditions in which microbial signals, activities, or rhythmic patterns no longer remain aligned with host physiological regulation. A hallmarks-based framework is proposed to examine biological domains in which coordination between host regulation and microbial ecology deteriorates. Core hallmarks include breakdown of signaling fidelity, microbiome-driven immune miscalibration, barrier compartment failure, endocrine-microbiome uncoupling, ecological destabilization, and temporal desynchronization between host circadian programs and microbial oscillations. Additional dimensions include pathological microbial metabolite dominance with epigenetic embedding, endocrine and neuro-microbiome regulatory uncoupling, ecological destabilization of microbiome functional capacity, and temporal desynchronization between host circadian programs and microbial oscillations. Across inflammatory, metabolic, neurodegenerative, and neoplastic conditions, microbial activity may operate outside normal ecological constraints, influencing immune regulation, metabolic signaling, neuroimmune communication, and tumor-associated processes. Within this framework, resilience, signaling proportionality, host responses appropriately scaled to microbial input, and temporal coordination represent central properties of host-microbiome compatibility.

## Introduction

Microbial communities colonizing mucosal and epithelial surfaces form part of the biological environment within which host physiological processes operate. Advances in sequencing technologies and integrative multi-omics have improved the characterization of microbial genes, metabolites, and ecological dynamics across multiple anatomical sites, supporting the view that microbial communities function as components of the host biological environment rather than as external ecological entities. Analyses conducted within the Integrative Human Microbiome Project illustrated how microbial genes, metabolites, and ecological dynamics interact with host physiological processes across different anatomical sites and environmental contexts (Integrative Hmp (IHMP) Research Network Consortium, 2019; Ma et al., 2024). Conceptual syntheses in microbiome research similarly describe the human organism as a host-associated ecosystem in which microbial populations participate in metabolic activity, immune regulation, and epithelial stability within a broader host-microbe system (Rackaityte and Lynch, 2020).

Host-associated microbial ecosystems influence regulatory processes that extend across interconnected physiological systems. Communication between microbial communities and host immune systems contributes to the regulation of physiological processes throughout life (Zheng et al., 2020a). Similar interactions occur across interconnected organ systems, where microbial activity in one location may influence host physiology elsewhere. Within this context, host-microbe symbiosis describes coordinated relationships in which host regulatory environments and microbial ecological behavior remain aligned under physiological conditions (Anand and Mande, 2022).

As microbiome research has expanded, the term dysbiosis has frequently been used to describe microbial configurations associated with disease. In most formulations, dysbiosis refers to shifts in microbial composition, diversity, or abundance relative to patterns observed in healthy populations. While the concept has facilitated identification of microbial signatures across multiple conditions, several limitations have been recognized. Microbial community composition varies substantially across individuals and environments, making it difficult to define a universal microbial baseline (Safarchi et al., 2025; Gilbert and Lynch, 2019). Functional redundancy within microbial ecosystems further complicates interpretation, since distinct microbial configurations may support comparable ecological or metabolic functions.

Interpretation of dysbiosis is also influenced by methodological considerations. Many studies describe associations between microbial features and disease phenotypes without establishing temporal directionality, leaving open whether microbial changes precede, accompany, or follow host physiological alterations (Metwaly et al., 2025). Longitudinal investigations additionally indicate that microbial ecosystems exhibit adaptive properties such as resilience and ecological recovery, suggesting that microbial states may change over time rather than remain fixed configurations (Zhou et al., 2025). Observations of this type indicate that compositional descriptions alone may provide an incomplete representation of host-microbiome relationships.

Several recent frameworks therefore propose interpreting the microbiome within broader systems-level perspectives that integrate microbial ecology with host regulatory biology. Such approaches encourage examination of how host regulatory environments and microbial communities interact as components of a shared biological system. From this viewpoint, disturbances in host-microbiome relationships may arise not only from alterations in microbial composition but also from changes in the coordination between host regulatory processes and microbial ecological responses (Sabih Ur Rehman et al., 2025).

Host-microbiome decoupling is defined as a state in which microbial activities, functions, spatial distribution, or temporal dynamics no longer remain proportionately aligned with host sensing, containment, and regulatory response. The minimal criterion for decoupling is not taxonomic disturbance alone but a persistent mismatch between microbial behavior and host regulatory capacity. Such mismatch may arise when microbial functional outputs operate outside the regulatory buffering capacity of the host, when microbial rhythms lose phase coherence with host physiological programs, when spatial containment fails, or when host responses become disproportionate to otherwise ordinary microbial signals. In this sense, decoupling refers to impaired regulatory alignment within the host-microbiome system rather than to compositional change alone.

Multiple related terms are used to describe microbiome-associated disease states and therefore require conceptual clarification. Dysbiosis generally refers to alterations in microbial composition, diversity, or abundance relative to reference populations, whereas decoupling describes failure in the functional relationship between microbial ecosystems and host regulation. Loss of resilience indicates reduced ecological recovery following perturbation, functional instability reflects fluctuating or unreliable ecosystem performance, and pathobiont expansion denotes the proliferation of opportunistic members under permissive ecological conditions. Each process may contribute to host-microbiome decoupling, but none alone is sufficient to define it. The concept instead describes conditions in which host regulatory systems no longer maintain microbial activity within appropriate physiological, spatial, or temporal bounds.

A systems-level interpretation of host-microbiome interactions aligns with descriptions of the human organism as a host-microbe meta-system in which biological properties emerge from continuous interaction between host and microbial components. Interpretation of disease-associated microbial patterns therefore benefits from conceptual models capable of examining how coordination between host regulation and microbial ecology changes across physiological and pathological contexts (Bautista et al., 2026a; Fang et al., 2024).

**FIGURE 1:**
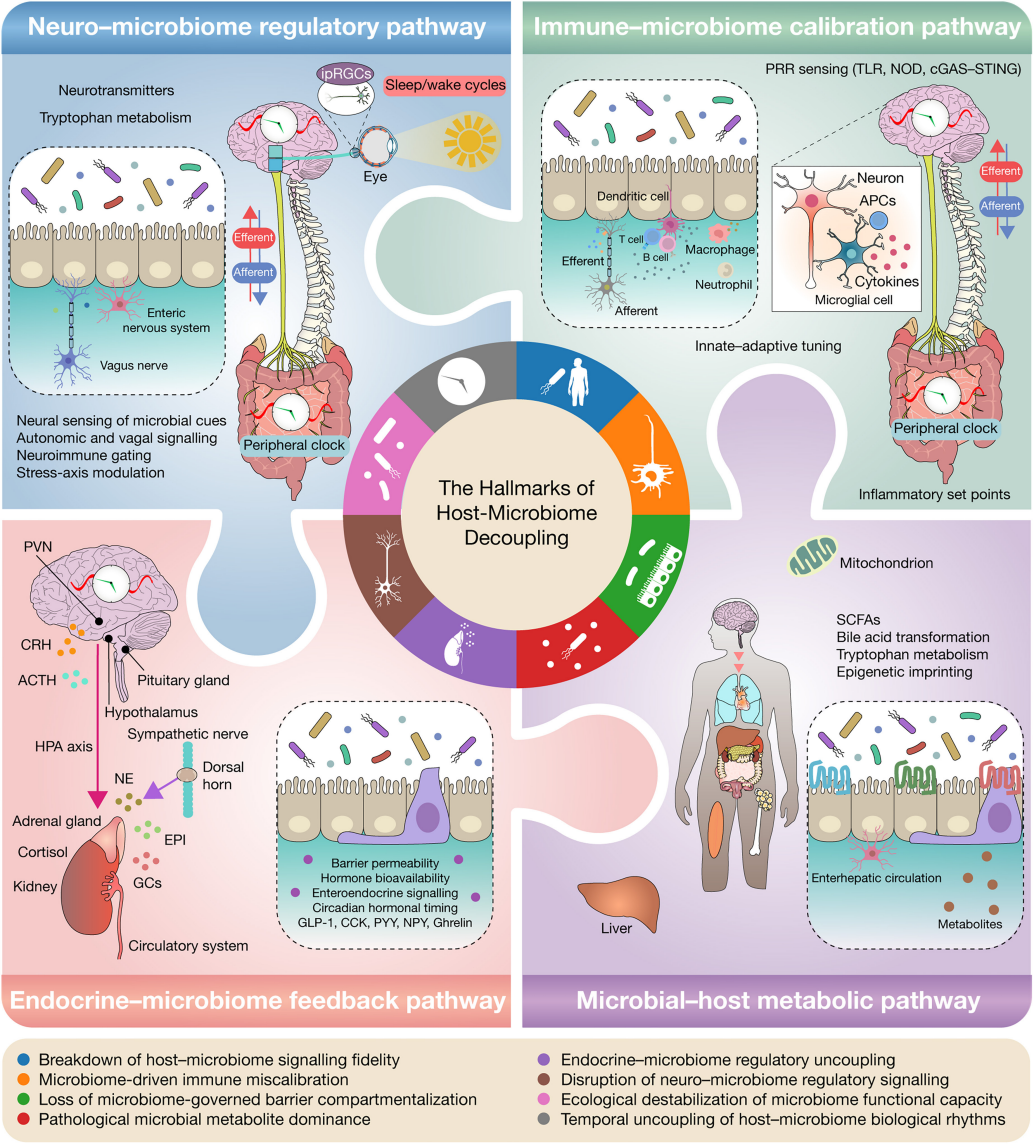
The hallmarks of host-microbiome decoupling across four higher-order regulatory axes. This figure presents a systems-level framework in which host-microbiome coordination is organized across four interlinked physiological axes: neuro-microbiome regulatory signaling, immune-microbiome calibration, endocrine-microbiome feedback, and microbial-host metabolic integration. Under homeostatic conditions, microbial communities provide context-dependent signals that calibrate immune set points, maintain epithelial compartmentalization, modulate neuroendocrine outputs, and synchronize circadian and metabolic programs. Within and across these axes, host-microbiome decoupling emerges through recurrent mechanistic hallmarks, including breakdown of signaling fidelity, microbiome-driven immune miscalibration, loss of microbiome-governed barrier compartmentalization, pathological microbial metabolite dominance, endocrine-microbiome regulatory uncoupling, disruption of neuro-microbiome regulatory signaling, ecological destabilization of microbiome functional capacity, and temporal uncoupling of host-microbiome biological rhythms. The four pathways therefore define the major domains of host-microbiome integration, whereas the hallmarks define the mechanistic modes through which this coordination progressively fails. Together, these processes contribute to chronic inflammatory, metabolic, neurodegenerative, and neoplastic disease states.

Analysis of host-microbiome relationships benefits from an organizational structure that captures how regulatory alignment between host physiology and microbial ecology progressively weakens. The hallmarks framework proposed here delineates biological domains in which this integration may deteriorate, extending interpretation beyond compositional descriptions of microbial communities. Each hallmark corresponds to a functional dimension of host-microbiome interaction whose disruption may affect physiological stability. These dimensions include signaling fidelity (accurate contextual interpretation of microbial signals by host sensing systems), proportionality (host responses scaled to microbial input), endocrine noise (irregular or poorly buffered hormone-related signaling arising from disrupted host-microbiome endocrine feedback), and ecological destabilization (loss of microbiome functional stability and resilience following perturbation). The following sections examine each hallmark and discuss their relevance across pathological contexts (Figure 1).

## Breakdown of host-microbiome signaling fidelity

Under physiological conditions, host-microbiome communication relies on tightly regulated sensing systems that discriminate between symbiotic microbial cues and genuine danger signals. Pattern recognition receptors (PRRs), including Toll-like receptors, NOD-like receptors, and cytosolic DNA sensors, are calibrated to maintain immune tolerance while preserving readiness against pathogens. Loss of host-microbiome signaling fidelity arises when microbial cues are no longer interpreted within their appropriate ecological and physiological context, resulting in disproportionate activation or suppression of downstream pathways that destabilize mucosal and systemic homeostasis (Al-Taweel et al., 2025; Di Vincenzo et al., 2024).

A prominent axis of signaling decoupling involves dysregulated nucleic acid sensing through the cGAS–STING pathway. Basal activation of cGAS–STING by microbiota-derived DNA contributes to immune vigilance and antiviral preparedness under homeostatic conditions. However, excessive or mislocalized activation drives pathological type I interferon signaling and sustained NF-κB–mediated inflammation. Aberrant delivery of bacterial DNA through extracellular vesicles or epithelial barrier disruption amplifies cytosolic sensing beyond physiological thresholds, transforming a protective surveillance pathway into a driver of persistent inflammatory activation (Yang et al., 2023; Erttmann et al., 2022).

Secretory immunoglobulin A (sIgA) represents another critical layer of signaling fidelity by shaping microbial behavior without eliciting inflammation. Through selective coating of commensal bacteria, sIgA restrains microbial encroachment, modulates microbial gene expression, and preserves spatial segregation between luminal microbes and the epithelium (Pietrzak et al., 2020). Quantitative loss or qualitative alteration of sIgA responses permits excessive microbial-epithelial contact and heightened engagement of PRRs, shifting immune-microbial interactions toward sustained inflammatory signaling (Ghosh and Sinha, 2025).

Barrier-associated signaling systems further contribute to host-microbiome decoupling when compromised. The mucus layer and antimicrobial peptides establish a biochemical and spatial buffer that limits microbial access while transmitting controlled microbial signals to epithelial and immune cells. Disruption of mucus architecture or antimicrobial peptide expression collapses this signaling filter, exposing epithelial sensors to high concentrations of microbial ligands. The resulting signal overload diminishes specificity and amplifies inflammatory cascades, undermining the host’s capacity to mount graded and proportional responses (Dupont et al., 2014; Ma et al., 2024).

Chronic low-grade inflammation driven by dysbiosis further degrades signaling fidelity by altering host responsiveness to microbial metabolites and structural components. Elevated circulating markers such as lipopolysaccharide, zonulin, and pro-inflammatory cytokines reflect a state in which microbial signals bypass regulatory checkpoints. Within this environment, immune cells mount non-selective responses that amplify systemic inflammation and sustain maladaptive feedback loops linking microbial perturbation to immune dysfunction (Peña-Durán et al., 2025; Di Vincenzo et al., 2024).

In oncological contexts, signaling decoupling acquires additional complexity. Tumor-associated microbiota can hijack host signaling pathways, activating oncogenic cascades, suppressing antitumor immunity, or reshaping the tumor microenvironment through aberrant PRR and cytokine signaling. Microbial metabolites and structural components engage host signaling circuits outside their original ecological context, facilitating immune evasion, therapeutic resistance, and tumor-promoting inflammation (Chen et al., 2023; Jiménez-Gracia et al., 2026).

Neural signaling represents an additional layer through which signaling fidelity is distorted in cancer. Tumor-derived neurotrophic cues can activate sensory and autonomic circuits that release immunomodulatory neuropeptides, including calcitonin gene-related peptide (CGRP) and substance P, which reshape immune cell recruitment, cytokine polarization, and checkpoint-associated T-cell phenotypes. Experimental models demonstrate that CGRP-RAMP1 signaling promotes T-cell exhaustion and limits the efficacy of immune checkpoint blockade, whereas pharmacological or genetic disruption of this axis restores antitumor immunity (Huang et al., 2025; Bautista et al., 2026d).

Beyond immune and neuroimmune modulation, intratumoral bacteria can directly reprogram tumor cell-intrinsic behavior. Microorganisms residing within malignant cells have been shown to enhance actomyosin contractility, focal adhesion dynamics, and cytoskeletal tension, thereby increasing cellular motility and invasive capacity. This mechanically driven reprogramming facilitates intravasation and metastatic dissemination without necessarily accelerating primary tumor growth, indicating a selective effect on migratory phenotypes rather than proliferative programs (Bautista et al., 2025b). At the molecular level, bacterial signals intersect with host cytoskeletal regulators and mechanotransduction pathways, effectively bypassing canonical growth-control checkpoints. Such findings highlight how microbial-derived cues can be co-opted into context-independent pro-migratory signals, extending the concept of signaling decoupling to tumor cell-autonomous programs that favor metastatic competence (Wang N. et al., 2025; Huang et al., 2025).

Overall, breakdown of host-microbiome signaling fidelity reflects a collapse of adaptive co-regulation within the meta-host system. When microbial inputs are no longer integrated as graded extensions of host physiology but instead act as persistent stressors, homeostatic signaling is replaced by chronic activation, loss of proportionality, and maladaptive feedback. This hallmark mechanistically links epithelial dysfunction, immune miscalibration, metabolic inflammation, and multisystem disease susceptibility, underscoring the need for therapeutic strategies aimed at restoring signaling integrity rather than solely modifying microbial composition (Sanam et al., 2025; Wang Q. et al., 2025).

## Microbiome-driven immune miscalibration

A central hallmark of host-microbiome decoupling is the progressive loss of immune calibration driven by altered microbial cues. Under homeostatic conditions, commensal microorganisms provide continuous, low-intensity signals that educate both innate and adaptive immunity, enabling discrimination between innocuous antigens and genuine threats (Heidari et al., 2024). When these inputs become chronically distorted in magnitude, timing, or composition, immune regulatory set points shift toward maladaptive states characterized by sustained inflammation, impaired antiviral defense, or inappropriate immune activation (Ignacio et al., 2024; Jain, 2020).

Immune calibration is established early in life through tightly regulated interactions between microbial colonization and immune ontogeny. Strain-resolved metagenomic analyses indicate that this process is strongly shaped by maternal microbial transmission. Vaginal delivery enables vertical inheritance of maternal gut and vaginal strains enriched in immunostimulatory functions, including lipopolysaccharide biosynthesis, which correlates with heightened neonatal TNF-α and IL-18 responses during critical windows of immune education. In contrast, cesarean delivery disrupts strain inheritance, attenuating early innate immune priming and predisposing to long-term immune miscalibration (Bautista and López-Cortés, 2025c). Disruption during these developmental windows establishes durable immune “set points” that remain resistant to correction later in life, predisposing hosts to exaggerated or misdirected immune responses (de Groen et al., 2026; Ignacio et al., 2024).

At the cellular level, microbiome-driven immune miscalibration is prominently manifested as an imbalance between regulatory and effector T-cell programs. Commensal-derived signals normally promote the differentiation and stability of FoxP3^+^ regulatory T cells while constraining pro-inflammatory Th17 responses. Dysbiosis alters microbial metabolite availability and antigenic landscapes, skewing this balance toward sustained Th17 polarization and impaired regulatory control. This shift establishes a chronic inflammatory tone rather than an acute defensive response, increasing susceptibility to autoimmune and inflammatory diseases (de Groen et al., 2026). Clinical and experimental evidence across immune-mediated diseases further demonstrates that altered microbial composition and metabolite profiles reinforce regulatory T-cell dysfunction while amplifying IL-17, and IFN-γ, dominated inflammatory circuits (Chen and Tang, 2021).

Innate immune miscalibration represents an equally critical dimension of this hallmark. Pattern-recognition receptors are normally tuned to interpret microbial ligands within a narrow physiological range that preserves immune proportionality. Dysbiotic ecosystems disrupt this tuning, promoting persistent activation of innate sensing pathways and sustained cytokine production. High-resolution single-cell and spatial transcriptomic analyses reveal that circulating immune cells in inflammatory and neoplastic states display conserved transcriptional signatures of dysregulated antigen presentation, interferon signaling, and cytokine responsiveness, consistent with chronic immune overactivation driven by altered microbial cues (Singh et al., 2025; Tufail et al., 2025).

Beyond surface receptor pathways, microbiome-dependent modulation of cytosolic immune sensors further contributes to immune miscalibration. Under physiological conditions, tonic microbial engagement of intracellular sensing pathways supports basal antiviral preparedness. Disruption of microbial communities attenuates this tonic signaling, increasing susceptibility to viral infection, whereas excessive or ectopic activation promotes immune exhaustion and inflammatory pathology (Seok et al., 2023). This bidirectional vulnerability highlights the narrow regulatory window required for microbiome-dependent interferon priming and underscores immune miscalibration as a consequence of signal imbalance rather than simple immune activation or suppression (Kratou et al., 2024; Tepekule et al., 2025).

## Loss of microbiome-governed barrier compartmentalization

Microbiome-governed barrier compartmentalization refers to the capacity of host-microbial ecosystems to maintain a spatially organized separation between luminal microorganisms, epithelial interfaces, immune compartments, and systemic circulation. Under homeostatic conditions, this compartmentalization is sustained by a multilayered barrier system comprising mucus and antimicrobial peptides, an intact epithelial monolayer sealed by tight junctions, controlled antigen sampling, and immune tolerance mechanisms that collectively prevent excessive microbial translocation while preserving metabolic exchange. Disruption of this finely tuned architecture represents a defining feature of host-microbiome decoupling, in which microbial ecological alterations and host barrier dysfunction converge to collapse spatial containment (Shin and Kim, 2018; Di Vincenzo et al., 2024).

Importantly, barrier compartmentalization can be conceptualized as a coupled, multi-boundary system extending beyond the intestinal epithelium to include the blood-brain barrier as a second critical immune gate. Evidence indicates that intestinal permeability elevates circulating microbial-associated molecular patterns, primes systemic innate immunity, and increases immune trafficking across the blood-brain barrier, thereby converting gut barrier failure into neuroimmune barrier stress. This dual-barrier dysfunction amplifies inflammatory spillover, facilitates immune cell access to the central nervous system, and reinforces host-microbiome decoupling as a system-wide failure of spatial governance rather than a gut-restricted defect (Bautista et al., 2025a; Bibolar et al., 2025).

A central consequence of compartmentalization loss involves increased intestinal permeability, permitting luminal microbial products, particularly endotoxins including lipopolysaccharide, to enter systemic circulation. Clinical evidence indicates that elevated intestinal permeability and metabolic endotoxemia are independently associated with increased cardiovascular mortality, supporting a model in which barrier failure operates as an upstream driver of systemic inflammatory disease rather than a passive byproduct. The evidence further indicates that microbiome-barrier uncoupling carries prognostic relevance beyond gastrointestinal pathology, extending into cardiometabolic risk (Parantainen et al., 2025; Bautista et al., 2025c).

Mechanistically, microbiome-induced permeability changes arise from coordinated structural and functional alterations in epithelial junctional complexes. Experimental models of obesity and diabetes demonstrate that specific gut microbial configurations actively promote disruption of epithelial tight junctions, increasing paracellular permeability and mucosal inflammation through defined molecular pathways rather than non-specific epithelial injury (Mishra et al., 2023). A microbiota-driven model of barrier breakdown reinforces the concept that microbial communities actively govern epithelial compartmentalization integrity (Chigozie et al., 2025).

The barrier is further destabilized by ecological perturbations that alter microbial resilience and functional output. Broad-spectrum antibiotic exposure induces long-lasting alterations across bacterial, phage, fungal, and metabolic layers of the gut ecosystem, with incomplete recovery even after apparent taxonomic normalization (d’Humières et al., 2024). Such ecological destabilization compromises microbial-derived signals essential for epithelial maintenance, weakening barrier resilience and predisposing the host to permeability shifts under secondary inflammatory or metabolic stress (Rose et al., 2025).

From a systemic perspective, impaired compartmentalization permits continuous leakage of microbial-associated molecular patterns into host circulation, sustaining chronic low-grade inflammation. In colorectal carcinogenesis, barrier collapse is frequently coupled to the formation of structured polymicrobial biofilms adherent to the colonic mucosa. These matrix-embedded consortia enable spatial and metabolic cooperation, including quorum sensing, horizontal gene transfer, and cross-feeding, and establish hypoxic inflammatory niches that maintain epithelial stress through persistent IL-6/STAT3 and NF-κB signaling while promoting immune evasion. The extracellular polymeric matrix further protects genotoxin-producing pathobionts from immune clearance and antimicrobial stress, amplifying tumor-promoting signaling within the local microenvironment (Wang et al., 2023; Bautista et al., 2026e). Reviews integrating clinical and experimental data indicate that increased intestinal permeability is tightly linked to systemic inflammatory activation across multiple organs, contributing to metabolic disease, immune dysregulation, and chronic inflammatory states. The resulting inflammatory spillover reflects a failure of spatial governance rather than isolated epithelial damage (Dmytriv et al., 2024).

Metabolic disease contexts provide a clear illustration of barrier-dependent immune dysregulation. In obesity and insulin resistance, barrier inefficiency facilitates endotoxin-driven inflammation that exacerbates metabolic dysfunction. Therapeutic strategies targeting barrier reinforcement through dietary modulation, microbiome-directed interventions, or metabolic regulation are increasingly recognized as viable approaches for restoring host-microbiome homeostasis. Available data position barrier integrity as a modifiable therapeutic axis rather than a fixed pathological outcome (Yu et al., 2025; Al-Busafi et al., 2025).

At the molecular level, barrier collapse is accompanied by coordinated alterations in epithelial junction proteins, immune mediators, and inflammatory markers. Reviews examining microbiota-inflammation interplay indicate that dysbiosis-driven permeability changes correlate with elevated pro-inflammatory cytokines and perturbed immune signaling, reinforcing a feed-forward interaction between microbial translocation and immune activation (Sun N. et al., 2024; Peña-Durán et al., 2025). Sustained bidirectional amplification accelerates compartmentalization failure by perpetuating epithelial stress and junctional instability (Shen et al., 2026).

Circulating biomarkers further substantiate the systemic impact of barrier breakdown. Clinical studies indicate that circulating endotoxin levels correlate strongly with intestinal epithelial injury markers, including fatty acid-binding protein 2, alongside activation of NF-κB signaling in immune cells, supporting direct gut-derived microbial translocation across compartmental boundaries and activation of systemic inflammatory pathways. Biomarker-based readouts therefore provide measurable indicators of microbiome-governed barrier failure in human populations (Zhang et al., 2025; Chen et al., 2025).

Beyond the gut, barrier dysfunction permits bacterial components and, in certain contexts, viable bacteria to access extraintestinal tissues. Reviews addressing metabolic disease emphasize that increased permeability facilitates tissue colonization and immune activation, blurring traditional distinctions between commensal and pathogenic microbial niches. Erosion of spatial segregation amplifies inflammation and metabolic dysregulation, reinforcing disease progression. Barrier integrity also remains tightly linked to neuroimmune signaling. In disorders of gut-brain interaction, altered intestinal permeability is associated with aberrant sensory signaling, immune activation, and symptom generation, illustrating that compartmentalization failure disrupts not only physical boundaries but also intersystem communication (Vanuytsel et al., 2023; Grover et al., 2025).

Finally, comprehensive syntheses position the intestinal barrier as a pivotal regulator of health, inflammation, and cancer. Barrier disruption is increasingly recognized as an enabling factor for tumor-promoting inflammation and microbial-immune crosstalk within neoplastic microenvironments, particularly in gastrointestinal malignancies (Neurath et al., 2025). In this context, loss of microbiome-governed compartmentalization represents a permissive state for chronic inflammation, immune dysregulation, and oncogenic signaling. At the systemic level, this failure extends beyond epithelial interfaces through the formation of pre-metastatic niches. Tumor-derived extracellular vesicles actively remodel distant tissues prior to tumor cell arrival by inducing extracellular matrix reorganization, vascular permeability, immune cell recruitment, and organ-specific permissiveness. Anticipatory erosion of spatial and immunological constraints converts distant organs into receptive microenvironments, functionally mirroring barrier breakdown and reinforcing host-microbiome decoupling across metastatic axes (Bautista and López-Cortés, 2025b; de Visser and Joyce, 2023).

## Pathological microbial metabolite dominance

One dimension of host-microbiome decoupling involves the emergence of pathological microbial metabolite dominance. In this context, specific microbial metabolic products accumulate or circulate in patterns that are no longer proportionate to host regulatory signals, altering physiological signaling networks and disrupting systemic homeostasis. Rather than functioning within balanced metabolic exchange, microbial metabolites begin to influence host regulatory pathways in patterns that are no longer aligned with host physiological control. At the molecular level, several microbial metabolites interact with host receptor systems that translate microbial signals into transcriptional and cellular responses. Short chain fatty acids (SCFAs) signal through G-protein-coupled receptors such as GPR41 and GPR43 and may also inhibit histone deacetylases, thereby influencing chromatin accessibility and gene expression in epithelial and immune cells. Microbially transformed bile acids interact with FXR and TGR5, pathways involved in inflammatory tone, epithelial stress responses, and metabolic regulation. In parallel, microbial tryptophan-derived indoles bind the aryl hydrocarbon receptor (AhR), which participates in epithelial maintenance and mucosal immune programming. When microbial metabolite production becomes disproportionate to host regulatory conditions, these receptor-mediated pathways may transmit signals across immune, metabolic, and barrier-associated processes (Miyamoto et al., 2024; Han et al., 2023; Chang, 2024).

Altered SCFA profiles illustrate this shift. Changes in microbial community structure and substrate availability modify both the concentration and relative proportions of acetate, propionate, and butyrate. Such alterations affect host signaling networks that normally integrate microbial metabolic outputs with nutritional inputs and circadian regulation. Experimental observations indicate that abnormal SCFA exposure can modify immune signaling, neuroendocrine responses, and metabolic regulation when microbial metabolite production becomes disconnected from host dietary and ecological context (Sandhu and Radhakrishnan, 2025; O’Riordan et al., 2022).

A comparable imbalance occurs in bile acid metabolism. Intestinal microbes convert primary bile acids into secondary derivatives that interact with host receptors regulating metabolic and immune processes. Under conditions of ecological disturbance, microbial enzymatic activity shifts bile acid pools toward species associated with inflammatory signaling and epithelial stress. These altered bile acid environments affect transcriptional programs in intestinal and hepatic tissues while simultaneously modifying microbial ecological dynamics, reinforcing unstable host-microbe interactions along the gut-liver axis (Molinaro et al., 2018; Cai et al., 2022).

Perturbations in microbial tryptophan metabolism provide an additional example of metabolic imbalance during host-microbiome decoupling. Microbial communities generate indole derivatives that interact with AhR signaling pathways involved in mucosal regulation. Disruptions in microbial metabolic activity modify the spectrum of tryptophan-derived metabolites, altering epithelial responses and immune signaling patterns. Experimental models indicate that reduced production of specific indole metabolites is associated with increased epithelial permeability and inflammatory activation, illustrating how localized metabolic shifts can propagate systemic physiological consequences (Xia and Huang, 2026; Siddiqui et al., 2025).

Microbial metabolites also influence host gene regulation through epigenetic mechanisms. Several microbial products interact with pathways involved in chromatin remodeling, DNA methylation, and transcriptional regulation. Changes in microbial metabolic output can therefore modify host transcriptional programs beyond immediate signaling responses. Experimental studies have shown that microbial metabolites including SCFAs, bile acids, and lipopolysaccharides interact with metabolic cofactors and epigenetic enzymes, contributing to durable transcriptional alterations in inflammatory and metabolic conditions (Tang et al., 2024; Guo et al., 2025; Rubas et al., 2025).

Because microbial metabolites circulate beyond the intestinal environment, metabolic disturbances associated with host-microbiome decoupling may affect multiple organ systems. Altered metabolite profiles have been detected in systemic circulation and linked to signaling changes in the liver, brain, and peripheral immune compartments. Along the gut-brain axis, shifts in microbial metabolic activity influence neuroimmune communication and stress-related signaling pathways. In parallel, metabolic products generated within intestinal microbial ecosystems can contribute to inflammatory and metabolic disturbances along the gut-liver axis (Yang et al., 2026; Sun X. et al., 2024).

## Endocrine-microbiome regulatory uncoupling

Endocrine-microbiome regulatory uncoupling represents a critical hallmark of host-microbiome decoupling, characterized by the progressive loss of bidirectional hormonal calibration between microbial ecosystems and host endocrine axes. Under physiological conditions, the gut microbiome acts as a distributed endocrine organ, modulating hormone synthesis, metabolism, receptor sensitivity, and feedback dynamics across multiple systems, including thyroid, estrogenic, metabolic, and stress-responsive axes. Disruption of this tightly regulated network results in endocrine noise, maladaptive hormone signaling, and systemic pathophysiology (Williams et al., 2020; Chao J. et al., 2025).

One of the most robust examples of endocrine-microbiome coupling is the gut-thyroid axis. Gut microbes regulate iodine bioavailability, selenium metabolism, and enterohepatic cycling of thyroid hormones through microbial deconjugation enzymes such as β-glucuronidases and sulfatases. Dysbiosis alters thyroid hormone bioavailability, impairs levothyroxine absorption, and perturbs hypothalamic-pituitary-thyroid (HPT) feedback loops, contributing to autoimmune thyroid disorders and metabolic instability. Loss of microbial control over peripheral thyroid hormone metabolism disrupts epithelial homeostasis and systemic energy regulation, exemplifying endocrine uncoupling at both local and organismal levels (Jiang et al., 2022; Fenneman et al., 2023).

Similarly, estrogen homeostasis is highly dependent on the functional integrity of the estrobolome, the subset of gut microbes capable of metabolizing estrogens and phytoestrogens. Microbial β-glucuronidase activity governs estrogen deconjugation and reabsorption, shaping circulating estrogen pools. Alterations in estrobolome composition reduce hormonal buffering capacity, leading to estrogen dominance or deficiency depending on ecological context. Such uncoupling has been implicated in estrogen-driven diseases, including endometriosis, menopausal metabolic disorders, and hormone-dependent cancers. Importantly, estrogen-microbiome uncoupling not only affects systemic hormone levels but also alters immune activation, inflammatory tone, and tissue-specific hormone responsiveness (Nannini et al., 2025; Larnder et al., 2025).

Enteroendocrine cells (EECs) serve as a central interface between luminal microbial signals and host endocrine responses. These specialized epithelial cells sense microbial metabolites, bile acids, and SCFAs, translating microbial cues into hormonal outputs such as GLP-1, PYY, serotonin, and ghrelin. Microbiome-driven modulation of EEC maturation, hormone vesicle trafficking, and receptor signaling enables precise coordination of appetite, glucose metabolism, gut motility, and gut-brain communication (Chao J. et al., 2025; Grundeken and El Aidy, 2025). Endocrine-microbiome uncoupling emerges when dysbiosis alters metabolite profiles, impairing EEC responsiveness and distorting hormonal signaling fidelity.

Stress-responsive endocrine pathways are particularly vulnerable to microbiome decoupling. The hypothalamic-pituitary-adrenal (HPA) axis is bidirectionally regulated by gut microbes through immune modulation, vagal signaling, and microbial metabolite production. Under chronic stress or dysbiosis, elevated glucocorticoid levels disrupt microbial ecology, increase gut permeability, and promote systemic inflammation, creating a self-reinforcing loop of endocrine and microbial dysregulation. This breakdown of microbial constraint over HPA axis activity contributes to neuroendocrine disorders, mood dysregulation, and metabolic disease (Rusch et al., 2023; Bertollo et al., 2025).

Crucially, endocrine-microbiome uncoupling is not limited to isolated hormonal pathways but reflects a systems-level failure of hormonal integration. Microbial regulation of bile acid signaling via FXR and TGR5, serotonin biosynthesis via enterochromaffin cells, and insulinotropic signaling through incretin hormones collectively orchestrates metabolic homeostasis. Dysbiosis-induced alterations in these pathways generate asynchronous hormonal signals that undermine circadian alignment, immune tolerance, and metabolic flexibility (Stanimirov et al., 2025; Grasa-Ciria et al., 2025).

Across endocrine axes, a unifying feature of uncoupling is the disruption of hormonal modulation. Rather than reflecting absolute hormone deficiency or excess, endocrine-microbiome decoupling manifests as inappropriate timing, localization, and amplitude of hormonal signals, increasing endocrine noise, weakening feedback inhibition, and heightening susceptibility to chronic inflammatory, metabolic, and neoplastic diseases (Aref et al., 2024; Hoermann et al., 2022).

## Disruption of neuro-microbiome regulatory signaling

Neuro-microbiome regulatory signaling emerges from an integrated, multilevel communication network linking the gut microbiota, the enteric nervous system (ENS), peripheral immunity, and central nervous system (CNS) circuits. Under physiological conditions, this axis enables microbial communities to calibrate neurodevelopment, synaptic plasticity, stress responsiveness, and neuroimmune homeostasis. Effects are mediated through tightly regulated neural, endocrine, metabolic, and immune pathways. Disruption of the underlying regulatory architecture constitutes a core hallmark of host-microbiome decoupling, wherein microbial signals lose contextual specificity and instead drive maladaptive neuroinflammatory and neurodegenerative programs (Ullah et al., 2023; Geng et al., 2022; Loh et al., 2024).

A central node within this signaling network is the ENS. Beyond its role as an autonomous neural system governing gut motility and secretion, the ENS functions as a bidirectional interface between microbial ecosystems and higher neural circuits. Experimental models demonstrate that gut microbial colonization actively shapes ENS maturation, neuronal renewal, and neurochemical output. These effects are largely mediated through serotonin-dependent mechanisms involving enterochromaffin cells and 5-HT4 receptor signaling (Zhuang et al., 2024). Loss of microbial input or dysbiosis impairs ENS plasticity, leading to altered intestinal transit, aberrant sensory signaling, and destabilized communication with the CNS. Conversely, ENS dysfunction perturbs microbial community structure, favoring pro-inflammatory taxa and perpetuating feed-forward loops of intestinal and systemic inflammation (Rolig et al., 2017).

Beyond the ENS, neuro-microbiome communication relies heavily on vagal afferent and efferent pathways that transmit microbial and immune-derived signals directly to brainstem nuclei. The vagus nerve integrates microbial metabolites, cytokine cues, and enteroendocrine signals to regulate mood, cognition, and stress resilience. Disruption of vagal signaling, whether driven by dysbiosis-associated inflammation or altered microbial metabolite profiles, has been causally linked to anxiety-like behavior, depressive phenotypes, and increased vulnerability to neurodegenerative processes (Kasarello et al., 2023; Abdel-Haq et al., 2019).

At the CNS level, microglia represent a critical downstream target of neuro-microbiome signaling. Microbiota-derived cues are required for proper microglial maturation, surveillance capacity, and transcriptional identity across development and adulthood. In germ-free or dysbiotic states, microglia adopt aberrant activation profiles characterized by impaired phagocytosis, exaggerated inflammatory responses, and defective synaptic pruning (Geng et al., 2022). These maladaptive microglial states contribute directly to neuroinflammation and neuronal dysfunction observed in Alzheimer’s disease, Parkinson’s disease, and autism spectrum disorders. Restoration of microbial-derived metabolites or partial reconstitution of microbial diversity rescues microglial homeostasis (Santos et al., 2025).

Neuro-microbiome decoupling is further amplified through immune-mediated pathways linking the gut and brain. The gut-brain axis operates in close coordination with innate and adaptive immune responses, with inflammasome activation, type I interferon signaling, and NF-κB pathways serving as key molecular conduits. Dysregulated microbial cues induce chronic peripheral inflammation that breaches neuroimmune barriers. This process facilitates immune cell trafficking into the CNS and promotes sustained neuroinflammatory states (Park et al., 2025). Resultant immune spillover disrupts blood-brain barrier integrity and reshapes cytokine gradients within the CNS, further decoupling neuronal circuits from homeostatic microbial inputs (Kasarello et al., 2023).

Metabolite-mediated signaling constitutes another critical layer of neuro-microbiome regulation. SCFAs, tryptophan derivatives, bile acid metabolites, and microbially derived neurotransmitters modulate neuronal excitability, glial activation, and neuroendocrine axes. Under dysbiotic conditions, metabolite profiles shift toward neurotoxic or pro-inflammatory states, impairing synaptic transmission and promoting oxidative stress within neural tissues (Loh et al., 2024). Loss of metabolite rhythmicity, normally synchronized with circadian and feeding cycles, disrupts temporal coordination between microbial activity and neural function and contributes to cognitive and behavioral instability (Zheng et al., 2025).

Temporal misalignment represents a fundamental yet underappreciated dimension of neuro-microbiome decoupling. The microbiota actively shapes circadian rhythms of immune and neural signaling through synchronized oscillations in microbial adherence, epithelial signaling, and innate immune activation. Disruption of these rhythms, whether driven by altered feeding patterns or microbial dysbiosis, results in desynchronized neuroimmune signaling and impaired host anticipation of environmental stressors (Bautista and López-Cortés, 2025a; Beurel, 2024; Han et al., 2022).

## Ecological destabilization of microbiome functional capacity

Ecological destabilization of microbiome functional capacity refers to the progressive loss of the microbiome’s ability to sustain core metabolic, immunomodulatory, and protective functions despite the continued presence of microbial biomass or apparent taxonomic diversity. This phenomenon reflects a decoupling between community structure and ecosystem-level performance, in which microbial functions become increasingly sensitive to perturbations and less capable of buffering host physiology against environmental and endogenous stressors (Lee et al., 2023; Fässler et al., 2025).

A foundational determinant of functional stability is functional redundancy, defined as the capacity of multiple phylogenetically distinct taxa to perform overlapping biochemical roles. Quantitative and network-based analyses demonstrate that redundancy is unevenly distributed across microbial traits and metabolites, and that its protective effect depends on interaction topology and interspecies dependencies rather than species richness alone. Consequently, communities with preserved diversity may nonetheless exhibit profound functional fragility when redundancy collapses within key metabolic pathways (Fässler et al., 2025; Li et al., 2024).

Mechanistically, functional destabilization may arise from disruption of distributed metabolic circuits rather than from simple loss of taxa. Many microbiome functions depend on cross-feeding interactions in which metabolites produced by one organism are further converted by neighboring taxa into bioactive end products. When keystone organisms decline, these cooperative pathways may become fragmented, leading to incomplete substrate conversion and accumulation of intermediate metabolites. Multi-omic analyses indicate that such fragmentation can occur even when overall microbial biomass or taxonomic diversity remains relatively stable, illustrating that ecosystem-level dysfunction may emerge from loss of network connectivity rather than from overt community collapse (Villette et al., 2025; Ramond et al., 2025).

Closely related to redundancy is microbiome resilience, the ecological property describing the ability of microbial communities to resist or recover from disturbances. While healthy adult microbiomes typically display high resilience, repeated or intense perturbations, such as antibiotics, inflammation, or dietary disruption, can push the system toward alternative stable states characterized by altered and often diminished functional output. Transitions may persist long after partial compositional recovery, indicating that functional destabilization can become entrenched even when taxonomic profiles appear normalized (Zhou et al., 2025).

Antibiotic exposure represents one of the most potent drivers of functional destabilization. Beyond reducing species diversity, antibiotics selectively eliminate metabolically active and keystone taxa, disrupting cooperative metabolic networks and impairing collective functions such as SCFA synthesis and bile acid transformation (Schwartz et al., 2020). Ecological and mathematical modeling reveals that post-antibiotic recovery frequently follows non-linear trajectories, with microbiomes stabilizing in functionally altered equilibria rather than returning to their original state, thereby reducing long-term buffering capacity (Shaw et al., 2019).

Functional destabilization also creates ecological niches that favor the expansion of pathobionts, opportunistic microbes that exploit weakened competition and impaired host-mediated compartmentalization. The overgrowth of organisms such as *Clostridioides difficile* exemplifies how loss of functional redundancy and immune calibration facilitates pathogenic dominance, amplifying inflammatory feedback loops that further suppress beneficial microbial activities (Petersen and Round, 2014; Chandra et al., 2021). In this context, destabilization represents a failure of ecosystem regulation rather than a simple overrepresentation of harmful taxa.

From a systems ecology perspective, destabilization is strongly influenced by the architecture of microbial interaction networks. Theoretical and empirical studies indicate that highly cooperative networks, while metabolically efficient, are inherently unstable when positive feedbacks dominate, allowing perturbations to propagate rapidly across the community. In contrast, moderated competition and constrained interactions enhance stability by limiting runaway dynamics. Host regulatory mechanisms, including immune exclusion, spatial structuring, and nutrient partitioning, are therefore critical for maintaining functional compartmentalization, and their disruption accelerates ecological failure (Chao Z. et al., 2025).

Conceptual critiques of the term “dysbiosis” emphasize that functional destabilization cannot be reliably inferred from static compositional snapshots. Large-scale longitudinal and multi-omics studies indicate that disease-associated microbiome shifts often account for only a limited fraction of functional variance, underscoring contributions from stochasticity, microbe-microbe interactions, and context-dependent gene expression. Such observations support framing destabilization as a dynamic, systems-level process rather than a fixed taxonomic state (Liu et al., 2025; Brüssow, 2020).

Emerging ecological frameworks introduce the concept of microbiome rescue, which prioritizes the restoration of lost functions rather than taxonomic reconstitution alone. Functional recovery may occur through dispersal, reactivation of dormant populations, horizontal gene transfer, or adaptive reorganization of metabolic networks. However, rescue capacity is constrained when functional redundancy has been irreversibly eroded or when selective pressures favor simplified yet maladaptive functional configurations, limiting the effectiveness of late-stage interventions (Shade, 2023).

## Temporal uncoupling of host-microbiome biological rhythms

Under physiological conditions, host circadian clocks and the gut microbiome form a tightly synchronized temporal network in which microbial composition, metabolic activity, and host regulatory pathways oscillate across the 24-h light-dark cycle. A substantial fraction of gut microbial taxa exhibit diurnal fluctuations in abundance, localization, and functional output, driven primarily by host-controlled feeding rhythms and peripheral clock gene expression rather than by passive responses to environmental light alone. These oscillations extend beyond taxonomic variation to encompass rhythmic microbial gene expression and metabolite production, enabling time-specific coordination of host metabolism, immunity, and endocrine signaling (Siebieszuk et al., 2023; Zheng et al., 2025).

Temporal uncoupling arises when this bidirectional synchronization is disrupted, resulting in a loss of phase coherence between host circadian programs and microbial oscillatory behavior. Modern lifestyle factors such as shift work, irregular feeding schedules, artificial light at night, and chronic sleep disruption profoundly alter microbial rhythmicity, leading to flattened or misaligned oscillations in both microbial composition and function (Lotti et al., 2023). Disruption of circadian timing not only dampens oscillations in microbial metabolites such as SCFAs but also destabilizes stress-axis rhythmicity, linking temporal dysbiosis to neuroendocrine miscalibration. Experimental evidence further indicates that circadian rhythm disruption can perturb intestinal epithelial clock programs (e.g., *Per2*), drive microbiota dysbiosis, and, via fecal microbiota transplantation (FMT), transmit depression-like phenotypes to naïve recipients, supporting a causal role for microbiota-clock misalignment in mood and stress vulnerability (Bishehsari et al., 2020). Consistent with these mechanistic findings, experimental and population-level studies demonstrate that circadian misalignment associates with impaired microbial rhythmicity, altered metabolic signaling, and increased disease susceptibility, supporting temporal dysbiosis as a distinct pathological state rather than a secondary feature of compositional imbalance (Zheng et al., 2025).

At the molecular level, host-microbiome temporal coordination is linked to transcriptional feedback loops involving core circadian regulators such as BMAL1, CLOCK, PER, and CRY. These transcription factors contribute to rhythmic expression of epithelial transporters, immune mediators, and antimicrobial programs that influence microbial nutrient availability and mucosal spatial organization. Microbial metabolites can in turn influence host clock gene expression through receptor-mediated and epigenetic mechanisms that affect peripheral clock activity in intestinal and hepatic tissues. When this reciprocal regulation becomes disrupted, oscillatory gene expression may lose phase coherence, producing temporally misaligned immune, metabolic, and barrier-related responses (Zhao et al., 2026; Bautista and Lopez-Cortes, 2025).

Mechanistically, feeding time functions as a dominant non-photic zeitgeber that entrains microbial rhythms independently of the central circadian pacemaker. Delayed or mistimed feeding disrupts diurnal oscillations in microbial diversity and taxon-specific abundance, even when photic cues and gut physicochemical parameters such as pH remain rhythmic (Melville et al., 2025). Available observations indicate that microbial temporal organization is actively programmed to align with host feeding-fasting cycles, optimizing nutrient utilization and metabolite delivery across circadian phases. Decoupling of feeding rhythms from host clocks leads to desynchronized microbial oscillations, impairing functional coordination with host tissues (Crespo et al., 2025; Zheng et al., 2020b).

At the molecular level, microbial metabolites act as critical temporal signals linking microbial activity to host clock machinery. SCFAs, secondary bile acids, and tryptophan-derived metabolites exhibit circadian oscillations that influence the expression and epigenetic regulation of core clock genes through receptor-mediated pathways such as GPR41/43, FXR, and AhR signaling (Tofani et al., 2025). Disruption of microbial rhythmicity alters the temporal availability of these metabolites, leading to aberrant clock gene expression in peripheral tissues, particularly the intestine and liver, thereby weakening systemic circadian coherence (Zhang et al., 2023).

Temporal uncoupling also exerts profound effects on immune regulation. The microbiota coordinates diurnal rhythms in innate immune defenses, including oscillations in antimicrobial peptide production and immune cell activation that anticipate daily fluctuations in microbial exposure. Rhythmic attachment of specific commensals drives time-dependent activation of epithelial STAT3 signaling and downstream immune effector programs. Loss of microbial rhythmicity abolishes these oscillations, resulting in time-of-day independent immune responses and increased vulnerability to infection and inflammatory pathology (Zheng et al., 2025; Schmid et al., 2023).

Neuroendocrine regulation constitutes another critical axis affected by temporal host-microbiome decoupling. Oscillatory dynamics within gut microbial communities modulate hypothalamic-pituitary-adrenal axis rhythmicity, shaping diurnal patterns of glucocorticoid release and stress responsiveness. Microbial depletion or loss of rhythmicity disrupts circadian transcriptional programs in stress-responsive brain regions and alters glucocorticoid dynamics, generating time-dependent impairments in adaptive stress responses. Maintenance of proper alignment between circadian and stress regulatory systems therefore depends on intact microbial temporal signaling (Tofani et al., 2025; Heddes et al., 2022).

Importantly, experimental manipulation of host circadian clocks demonstrates that microbial rhythmicity is not merely reactive but actively maintained by host clock function. Intestinal epithelial cell-specific disruption of core clock genes abolishes microbial oscillations and alters microbial metabolic output, including bile acid and fatty acid profiles. Transfer of arrhythmic microbiota into germ-free hosts recapitulates immune and metabolic dysregulation, confirming that temporal integrity of the microbiome is essential for gastrointestinal and systemic homeostasis (Heddes et al., 2022; Zhang et al., 2023).

## Limitations

Several limitations should be considered when interpreting host-microbiome decoupling as a conceptual framework for chronic disease. Direct causal evidence in humans remains limited for many of the biological pathways discussed in this review. Much of the available literature relies on cross-sectional microbiome profiling, associative cohort studies, or experimental systems that cannot fully reproduce the temporal and physiological complexity of human disease. Large integrative initiatives have generated detailed host-microbiome datasets, yet these resources primarily reveal patterns of co-variation rather than causal directionality between microbial activity and host physiology (Ma et al., 2024; Chen et al., 2026).

Measurement represents a second limitation. The hallmarks proposed here describe functional properties such as signaling fidelity, ecological stability, and temporal coordination. These processes are dynamic and cannot be adequately captured through taxonomic composition alone. Microbiome studies remain highly sensitive to sampling strategy, sequencing technology, bioinformatic pipelines, and statistical modeling. Methodological variation continues to influence reproducibility and complicates comparisons across cohorts. Translating these hallmarks into clinically measurable parameters will therefore require standardized multi-layered approaches that integrate microbial, metabolic, and host-derived measurements (Batarseh and Koskella, 2025; Knight et al., 2018).

Conceptual scope also requires caution. A hallmarks-based framework can facilitate integration across biological systems, yet there is a risk of excessive generalization if diverse mechanisms are grouped within a single explanatory model. Host-microbiome interactions are shaped by diet, host genetics, immune state, medication exposure, mucosal physiology, and environmental context. These factors may produce different microbial configurations and regulatory responses across diseases and tissues. Previous critiques of dysbiosis have shown that conceptual models lose explanatory precision when definitions extend beyond measurable biological boundaries (Corral López et al., 2026).

Interpretation of multi-omic associations introduces an additional challenge. Systems-level datasets frequently identify correlations between microbial functions, host pathways, metabolites, and clinical phenotypes. However, distinguishing causal mechanisms from host-driven ecological change remains difficult. Population-scale studies demonstrate substantial interindividual variability in microbiome composition and function, while intervention studies show that host responses to microbiome-directed therapies are often personalized and context dependent (Shahid, 2025; Bautista et al., 2026b).

Within these constraints, host-microbiome decoupling should be interpreted as a conceptual model intended to organize complex host-microbial interactions rather than as a definitive causal taxonomy of disease. Progress in this area will require longitudinal human studies, temporally resolved sampling, functional validation, and experimental designs capable of separating host-driven microbial shifts from microbiome-driven effects on host regulation (Vestergaard et al., 2026).

## Conclusion and future perspectives

The concept of host-microbiome decoupling provides a framework to examine how chronic disease may develop when microbial ecosystems and host regulatory systems no longer remain functionally aligned. Across the studies discussed in the present review, disease-associated alterations were not limited to compositional imbalance but also involved reduced ecological resilience, impaired barrier regulation, altered immune calibration, and loss of temporal coordination between microbial activity and host physiology (Shen et al., 2025; Ma et al., 2024).

Current evidence indicates that the biological relevance of host-microbiome disturbances does not depend solely on taxonomic instability but also on loss of context-dependent regulatory function. The role of microbial metabolites illustrates such dynamics. Effects of SCFAs, bile acid derivatives, and tryptophan metabolites depend on timing, concentration, receptor availability, and host physiological state. Under such conditions, regulatory signals that normally contribute to immune, metabolic, and neuroendocrine homeostasis may become misaligned with host demand, contributing to persistent dysfunction across tissues (Park et al., 2025; Peña-Durán et al., 2025). Developmental timing also appears relevant, as early-life microbial perturbations may alter immune and epithelial programming in ways that remain difficult to reverse later in life (Bautista and López-Cortés, 2025c).

A critical consideration is that the proposed hallmarks should not be interpreted as uniformly present, equally weighted, or mechanistically primary across all disease states. Their contribution likely varies according to tissue context, developmental timing, environmental exposure, and disease stage, consistent with evidence indicating that microbiome configurations and their physiological effects differ substantially across hosts and anatomical niches rather than conforming to a single reference state. In some conditions, barrier failure and immune miscalibration may precede broader ecological and metabolic instability, whereas in other contexts endocrine disruption, temporal desynchronization, or altered metabolite signaling may exert earlier constraints on host-microbiome coordination. Neoplastic environments introduce an additional layer of complexity, since several alterations may arise as consequences of tumor-associated remodeling of immunity, metabolism, and tissue architecture rather than as initiating events. Evidence supporting individual hallmarks also varies in strength, particularly when comparing mechanistic studies with longitudinal human data and considering the persistent difficulty of establishing causal relationships in microbiome research (Metwaly et al., 2025; Joos et al., 2025).

Several observations hold relevance for clinical translation. Microbiome-directed interventions are unlikely to produce uniform outcomes when applied without consideration of host immune status, barrier integrity, ecological history, or circadian organization. Variable responses reported for fecal microbiota transplantation in immuno-oncology, together with more consistent associations observed for fiber-rich dietary patterns, illustrate that therapeutic effects may depend more on restoration of functional responsiveness than on microbial replacement alone (Bautista et al., 2025d; Kang and Cai, 2021; Davar et al., 2021). Longitudinal sampling and multi-omic profiling may therefore assist in identifying patient subgroups characterized by microbial resilience, signaling capacity, and susceptibility to ecological disruption rather than by static compositional traits alone (Ma et al., 2024; Bautista et al., 2026c).

Future studies should determine the conditions under which host-microbiome misalignment becomes sustained, biologically consequential, and therapeutically modifiable, and whether individual hallmarks function as initiating disturbances, downstream amplifiers, or context-dependent correlates across disease states. Addressing these questions will require experimental designs with temporal resolution, functional assays, and models capable of capturing bidirectional interactions between host regulatory networks and microbial ecosystems. In this context, host-microbiome decoupling can be examined not as a universal explanation for chronic disease but as a testable framework for evaluating when disruption of host-microbial coordination contributes to pathology and when restoration of regulatory alignment remains feasible (Di Vincenzo et al., 2024; Chao J. et al., 2025).

Early detection of host-microbiome decoupling will likely require study designs capable of capturing temporal and functional dynamics rather than single cross-sectional measurements. Longitudinal cohort studies with repeated sampling across microbial, metabolic, and immune layers provide an appropriate framework for examining progressive changes in host-microbial coordination. Within such designs, indicators such as ecological resilience after perturbation, stability of metabolite production, and preservation of microbial rhythmicity relative to host physiology may indicate early alterations in regulatory alignment before clear compositional disruption becomes detectable (Vestergaard et al., 2026).

Operational application of the hallmarks framework may also depend on integration of functional readout layers corresponding to distinct domains of host-microbiome regulation. Barrier-associated hallmarks can be examined through epithelial permeability measurements and mucosal immune profiling, metabolic hallmarks through quantification of microbial metabolites and host receptor signaling, and temporal coordination through analysis of microbial rhythmicity in relation to host circadian markers. Integration of these layers within multi-omic analytical approaches may allow patient stratification based on functional stages of host-microbiome coordination rather than taxonomic composition alone (Chetty and Blekhman, 2024; Zheng et al., 2025).
